# Mitochondrial unfolded protein response (UPR^mt^): what we know thus far

**DOI:** 10.3389/fcell.2024.1405393

**Published:** 2024-05-31

**Authors:** Angie K. Torres, Veronika Fleischhart, Nibaldo C. Inestrosa

**Affiliations:** ^1^ Facultad de Ciencias Biológicas, Pontificia Universidad Católica de Chile, Santiago, Chile; ^2^ Centro de Excelencia en Biomedicina de Magallanes (CEBIMA), Escuela de Medicina, Universidad de Magallanes, Punta Arenas, Chile

**Keywords:** *Caenorhabditis elegans*, mitochondria, UPR^mt^, stress, misfolded protein, wnt signaling

## Abstract

Mitochondria are key organelles for the optimal function of the cell. Among their many functions, they maintain protein homeostasis through their own proteostatic machinery, which involves proteases and chaperones that regulate protein import and folding inside mitochondria. In the early 2000s, the mitochondrial unfolded protein response (UPR^mt^) was first described in mammalian cells. This stress response is activated by the accumulation of unfolded/misfolded proteins within the mitochondrial matrix, which results in the transmission of a signal to the nucleus to increase the expression of proteases and chaperones to address the abnormal mitochondrial protein load. After its discovery, this retrograde signaling pathway has also been described in other organisms of different complexities, suggesting that it is a conserved stress response. Although there are some specific differences among organisms, the mechanism of this stress response is mostly similar and involves the transmission of a signal from mitochondria to the nucleus that induces chromatin remodeling to allow the binding of specific transcription factors to the promoters of chaperones and proteases. In the last decade, proteins and signaling pathways that could be involved in the regulation of the UPR^mt^, including the Wnt signaling pathway, have been described. This minireview aims to summarize what is known about the mechanism of the UPR^mt^ and its regulation, specifically in mammals and *C. elegans*.

## 1 Introduction

Mitochondria are organelles with many functions, such as providing energy in the form of ATP and regulating calcium homeostasis, redox balance, and apoptosis (Harrington et al., 2023), being essential for maintaining cellular homeostasis. Interestingly, mitochondria contain nuclear-encoded and mitochondrial-encoded proteins that are assembled inside the organelle to form functional complexes within the mitochondrial matrix and the inner mitochondrial membrane (Annesley and Fisher, 2019). Thus, mitochondria have a proteostatic network composed of chaperones and proteases that ensure correct protein import and folding within them (Voos, 2009). In 2002, the mitochondrial unfolded protein response (UPR^mt^) was described for the first time as a specific stress response in the mitochondria of mammalian cells triggered by the accumulation of unfolded/misfolded proteins within the mitochondrial matrix (Zhao et al., 2002); this type of response had been previously described only in the endoplasmic reticulum, known as the endoplasmic reticulum unfolded protein response (UPR^ER^).

The UPR^mt^ involves retrograde signaling between mitochondria and the nucleus, which leads to the upregulation of several mitochondrial proteins, including antioxidant enzymes, mitochondrial import proteins, and mitochondrial chaperones and proteases, to decrease the unfolded/misfolded load (Zhao et al., 2002; Anderson and Haynes, 2020; Tran and Van Aken, 2020). The mechanism of the UPR^mt^ in mammals has recently been described; however, it is still not fully understood. There are different axes of the UPR^mt^, with the canonical axis being the most studied (Munch, 2018); this axis involves the activation of the *integrated stress response* (ISR), which decreases the global translation rate, favoring the translation of specific stress-responsive proteins (Fiorese et al., 2016; Quiros et al., 2017; Anderson and Haynes, 2020). In the years after the UPR^mt^ was first described in mammalian cells, this stress response was also described in the nematode *C. elegans* (*C. elegans*) (Yoneda et al., 2004), in yeast (Schleit et al., 2013) and in *Drosophila melanogaster* (Pareek and Pallanck, 2018); this suggests that the UPR^mt^ is a conserved signaling pathway among eukaryotic organisms, including highly complex organisms such as humans and other mammals, and less complex organisms such as *C. elegans* and yeast. The mechanism of this stress response in *C. elegans* has been widely reported, more than the same response in mammals, mainly due to the ease of generating loss- or gain-of-function mutations in specific proteins in *C. elegans* (Haynes et al., 2007; Nargund et al., 2012); however, the mechanisms by which this stress response is regulated have not yet been fully described.

In this minireview, we will describe the mechanism of the UPR^mt^ both in mammals and in *C. elegans,* as well as the similarities and differences among these species, and the different regulatory mechanisms described in recent years to provide a global view of what is known about the UPR^mt^ today, providing a reference for future studies on the potential of this stress response as a new therapeutic target. The PubMed database was searched using the main keyword “*mitochondrial unfolded protein response*”, and articles describing the mechanism of the UPR^mt^ and reporting more recent findings regarding the regulation of the UPR^mt^ and its effect on diseases were selected.

## 2 The UPR^mt^ in mammals

**FIGURE 1:**
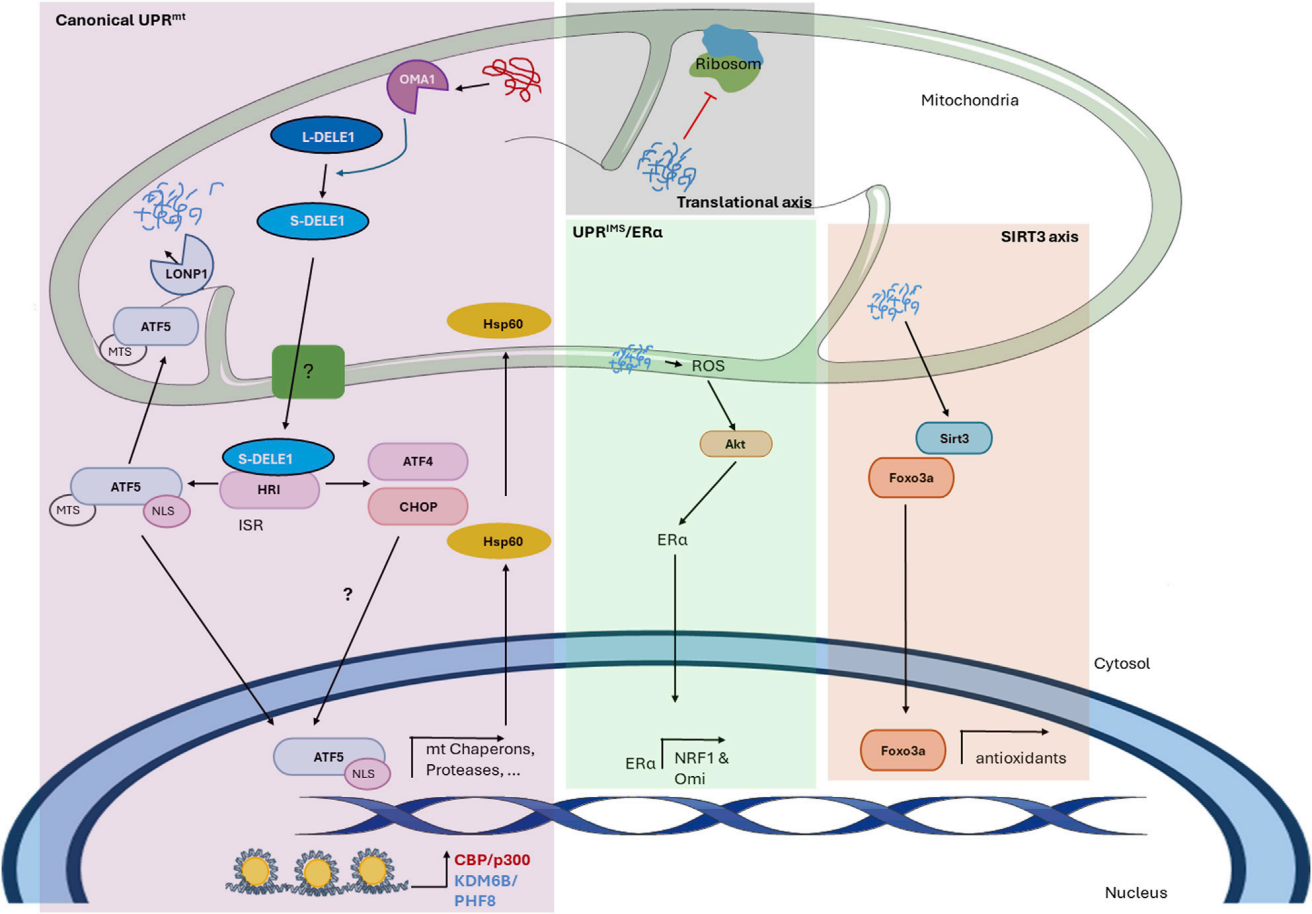
The mitochondrial unfolded protein response in mammals. The figure shows the mechanism of the UPR^mt^ in mammals described thus far. The scheme shows the different axes of the mitochondrial-nuclear retrograde signaling pathway and the proteins involved. In the *canonical UPR* ^*mt*^, the accumulation of abnormal proteins within the mitochondrial matrix activates the protease Oma1, which cleaves L-DELE1 into short fragments (S-DELE1) that are released into the cytosol. Once in the cytosol, S-DELE1 interacts with and activates the kinase HRI, activating the ISR and allowing the translation of ATF4, CHOP, and ATF5. The latter is translocated to the nucleus, where it binds to the promoters of UPR^mt^-related genes following chromatin remodeling by KDM6B and PHF8. CBP/p300 is also involved in epigenetic modification and the expression of UPR^mt^-related genes. In the *translational axis*, unfolded proteins reduce the mitochondrial translation rate locally without generating a global response. In the *SIRT3 axis*, unfolded proteins activate SIRT3, which induces the nuclear localization of the transcription factor FOXO3a to upregulate antioxidant enzyme expression. Finally, the *UPR* ^*IMS/*^ *ERα* is activated by misfolded protein accumulation in the IMS, leading to an increase in ROS levels, which in turn activates Akt kinase to phosphorylate and activate ERα. Undescribed proteins and mechanisms are shown with a question mark (?).

Regarding studies on the UPR^mt^ in mammals, Zhao et al. (2002) showed that the transfection of COS-7 cells with a mutant misfolded form of ornithine trans-carbamylase (ΔOTC), a mitochondrial matrix protein involved in the urea cycle, results in the accumulation of this protein, inducing the upregulation of nuclear-encoded mitochondrial chaperones and proteases (Zhao et al., 2002). This work described for the first time the transcriptional UPR^mt^, a stress response triggered by the accumulation of misfolded proteins within the matrix that is currently known as the canonical UPR^mt^ (Figure 1). In this stress response, stress signal transmission to the cytosol is thought to be driven through the processing of DAP3 binding cell death enhancer 1 (DELE1) by the protease Oma1 (Fessler et al., 2020; Guo et al., 2020). Oma1 constitutively cleaves the fusion dynamin-like GTPase L-OPA1 into small fragments, and this activity is increased under stress (Baker et al., 2014). Although the mechanism underlying stress-induced Oma1 activation is still not well characterized, it has been reported that stress signals are sensed through positively charged amino acids in the N-terminal region and that the transition to an active complex is associated with conformational changes involving the conserved C-terminal region (Baker et al., 2014). DELE1 is a 56 kDa protein that contains a mitochondrial targeting sequence (MTS) that allows it to be localized to the mitochondrial matrix in the absence of stress, where it has a short half-life due to its degradation by the protease Lonp1. However, during mitochondrial stress, DELE1 senses mitochondrial import deficiency since newly synthesized full-length DELE1 is cleaved by Oma1 in the N-terminal MTS, which produces short fragments (S-DELE1) that accumulate in the cytosol (Fessler et al., 2020; Guo et al., 2020). These fragments interact with and activate the kinase heme-regulated inhibitor (HRI), leading to the phosphorylation of eukaryotic translation initiation factor 2A (eIF2α), activating the ISR and increasing the expression of the transcription factors activating transcription factor 5 (ATF5), activating transcription factor 4 (ATF4) and C/EBP homologous protein (CHOP) (Fiorese et al., 2016; Fessler et al., 2020; Guo et al., 2020).

Although the order in which these transcription factors act is still unknown, ATF5 has been described as fundamental for UPR^mt^ activation. ATF5 has an MTS and a nuclear localization signal (NLS) (Fiorese et al., 2016). Under normal conditions, ATF5 is imported into mitochondria for degradation, presumably by the protease Lonp1. Nevertheless, under stress conditions, the import of ATF5 is inhibited by an unknown mechanism, leading to its cytoplasmic accumulation and consequent nuclear translocation, where it binds to a specific UPR^mt^ element to induce the expression of certain genes, including the mitochondrial chaperones Hsp60, Hsp10, and mtHsp70 and the proteases Lonp1 and ClpP (Nargund et al., 2015; Fiorese et al., 2016). Additionally, UPR^mt^-related genes contain CHOP-binding regions in their promoters, indicating the importance of CHOP in the expression of these genes (Zhao et al., 2002). CHOP induces ATF5 expression in the UPR^ER^ to induce apoptosis (Teske et al., 2013), and it has been reported that in HepG2 cells but not in other cells exposed to arsenite, ATF5 increases CHOP expression (Yamazaki et al., 2010), suggesting that these two transcription factors can regulate each other in a context-dependent manner. However, specifically in the UPR^mt^, the mechanism by which this process is regulated remains to be elucidated. Moreover, the precise role of ATF4 in this process is still unclear; however, it was identified as a regulatory factor that induces the expression of cytoprotective genes in response to mitochondrial stress, and it has also been proposed as a link between the UPR^ER^ and UPR^mt^ (Quiros et al., 2017; Jiang et al., 2020). Moreover, a previous study demonstrated that heat shock transcription factor 1 (HSF1) is an important player in the UPR^mt^ since under stress, HSF1 enters the nucleus and binds to the promoter of the chaperones Hsp60, Hsp10, and mtHsp70 but not to the promoter of the protease Lonp1 (Katiyar et al., 2020). However, how HSF-1 interacts with the canonical transcription factors ATF5, ATF4, and CHOP is still unknown.

It has been suggested that the UPR^mt^ involves epigenetic modifications caused by the histone demethylases PHF8 and KDM6B since there is a positive correlation between the expression of these proteins and UPR^mt^ gene expression. Furthermore, removal of lysine 27 trimethylation in histone 3 (H3K27me3) increases the expression of mitochondrial chaperones and proteases (Merkwirth et al., 2016). Moreover, the transcriptional coactivator *CBP/p300* induces the acetylation of lysine 18 and 27 in histone 3 (H3K18Ac and H3K27Ac), probably after KDM6B and PHF8 exert their effect, and is indispensable for UPR^mt^-related gene expression (Li et al., 2021).

Along with the canonical UPR^mt^, different axes of the UPR^mt^ have been described. There is a *translational axis* that decreases the mitochondrial translation rate locally to reduce the protein folding load and allow the handling of existing misfolded proteins (Munch and Harper, 2016). This translational axis is a local response and does not generate a cellular response since it is activated only when a few mitochondria are damaged (Munch, 2018). Moreover, the misfolded protein load in the mitochondrial matrix activates an *antioxidant UPR* ^*mt*^ *axis* driven by sirtuin 3 (SIRT3) (Papa and Germain, 2014; Munch, 2018). SIRT3 increases the nuclear localization of the transcription factor FOXO3a through its deacetylation, which increases the transcription of antioxidant enzymes such as superoxide dismutase 2 (SOD2) and catalase (Papa and Germain, 2014). Although it has been reported that Hsp10 and Lonp1 are substrates of SIRT3 deacetylation (Gibellini et al., 2014; Lu et al., 2015), there are contradictory findings regarding how the SIRT3 axis is related to canonical UPR^mt^-related gene expression (Gibellini et al., 2014; Papa and Germain, 2014; Lu et al., 2015; Chen et al., 2021; Wu et al., 2023). In addition, mitochondria have different compartments, and when misfolded protein accumulation occurs in the intermembrane space (IMS), another UPR, called the *UPR* ^*IMS*^ */ERα*, is activated. This signaling pathway seems to be independent of the canonical UPR^mt^; however, these responses can act in parallel or complement each other. Protein aggregates in the IMS activate estrogen receptor alpha (ERα) in a ligand-independent manner through its phosphorylation at serine 167 (Papa and Germain, 2011). An increase in reactive oxygen species (ROS) production leads to the activation of the kinase AKT, which ultimately induces the activation of ERα and the transcription of nuclear respiratory factor 1 (NRF1) and the IMS protease Omi (Papa and Germain, 2011). Altogether, these UPR^mt^ axes cope with the misfolded/unfolded load within mitochondria to maintain proper mitochondrial function.

## 3 The UPR^mt^ in *Caenorhabditis elegans*

**FIGURE 2:**
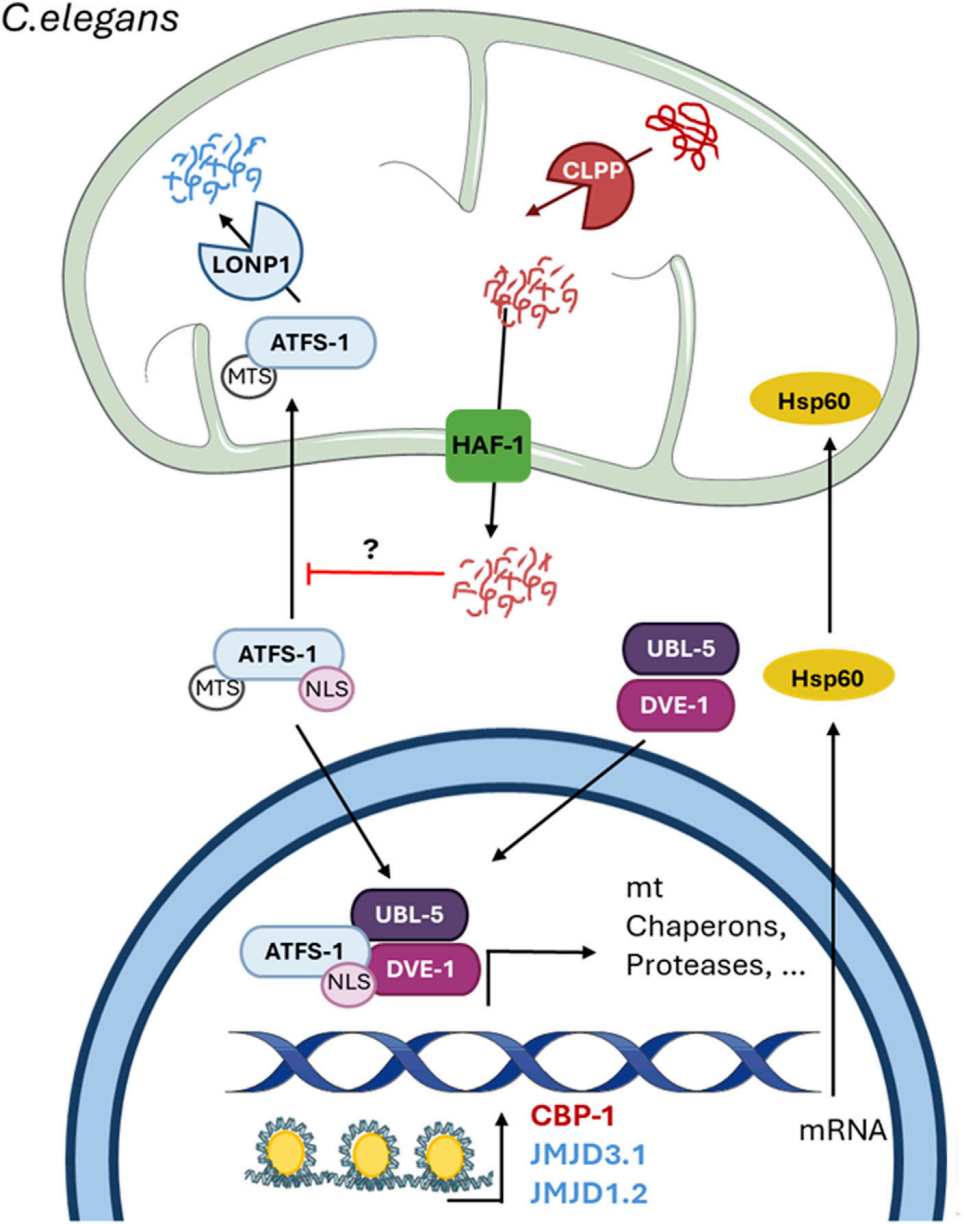
The mitochondrial unfolded protein response in *C. elegans*. The figure shows the mechanism of the UPR^mt^ in *C. elegans* described thus far. The scheme shows the mitochondrial-nuclear retrograde signaling pathway and the proteins involved. The protease ClpP cleaves abnormal proteins inside the mitochondria into small fragments of less than 20 amino acids, which enter the cytosol through the HAF-1 transporter. The release of these peptides inhibits the mitochondrial import of the transcription factor ATFS-1 by an unknown mechanism and induces its nuclear translocation. In the nucleus, ATFS-1 forms a complex with UBL-5 and DVE-1 after chromatin remodeling by the methyl transferases JMJD3.1 and JMJD1.2. Additionally, CBP-1 acts upstream of these two methyltransferases but downstream of ATFS-1 to induce the expression of UPR^mt^-related genes. Undescribed proteins and mechanisms are shown with a question mark (?).

Shortly after the UPR^mt^ was described in mammalian cells, it was reported that the perturbation of protein handling in mitochondria resulting from an RNAi against a mitochondrial protease induces the expression of mitochondrial chaperones (Yoneda et al., 2004). The mechanism of the UPR^mt^ in *C. elegans* has been largely described and, in general, is quite similar to that in mammals (Figure 2). Indeed, the signal produced by misfolded/unfolded accumulation in the matrix is transmitted to the cytosol by the release of short fragments, as in mammals; however, *C. elegans* does not have an Oma1 homolog (Kirstein-Miles and Morimoto, 2010). In *C. elegans*, the protease ClpP cleaves the abnormal proteins within the mitochondrial matrix into small fragments of approximately 20 residues that are released into the IMS through homodimers of the transporter HAF-1 in the inner mitochondrial membrane (IMM) (Haynes et al., 2010). The release of these peptides inhibits, by an unknown mechanism, the mitochondrial import of activating transcription factor associated with stress 1 (ATFS-1), an ATF5 homolog that is also the main transcription factor associated with the UPR^mt^ in *C. elegans.* Like ATF5, ATFS-1 contains a weak MTS and an NLS (Haynes et al., 2010); when mitochondria are not perturbed, the MTS prevails, and ATFS-1 is imported into the mitochondria for degradation by the protease LON. However, under mitochondrial stress, the weak MTS allows the sensing of few changes, which leads to inhibition of its mitochondrial import and consequent accumulation in the nucleus, where it can bind to the promoters of UPR^mt^-associated genes (Nargund et al., 2012). Additionally, a nuclear complex formed by the homeobox domain transcription factor DVE-1 and ubiquitin-like protein UBL-5 binds to the promoters of mitochondrial chaperones and protease to facilitate the subsequent binding of ATFS-1 (Haynes et al., 2007). The binding of these proteins to promoters requires chromatin remodeling, as in mammals. The accumulation of abnormal proteins activates the histone methyltransferase MET-2 and the nuclear localization of the cofactor LIN-65 (Tian et al., 2016). MET-2 mono- or dimethylates H3K9 (H3K9me1/2), which results in general chromatin remodeling, leaving specific regions exposed, where DVE-1 and ATFS-1 can bind (Tian et al., 2016). Along with these proteins, the histone deacetylase HAD-1 and the two histone demethylases JMJD-3.1 and JMJD-1.2 are fundamental for UPR^mt^-related gene expression (Merkwirth et al., 2016; Shao et al., 2020), indicating the importance of chromatin changes and epigenetics in the UPR^mt^. Furthermore, as in mammals, *the CBP/p300 homolog CBP-1* is necessary for UPR^mt^-related gene expression (Li et al., 2021). This coactivator is suggested to exert its effect between chromatin remodeling by JMJD-3.1 and JMJD-1.2 and the binding of ATFS-1 (Li et al., 2021).

Just like UPR^mt^ activation requires a decrease in global translation by the HRI-dependent activation of ISR in mammals, in *C. elegans* is also required a decrease in translation caused by phosphorylation of eIF2α, but in this case, by the kinase GCN2, which favors a better folding environment (Baker et al., 2012). These data support the idea that the UPR^mt^ and its mechanism are conserved between mammals and *C. elegans*.

## 4 The UPR^mt^ in disease

It has been reported that the UPR^mt^ is activated in different diseases in which mitochondrial dysfunction seems to be a key player, such as cardiac disease (Smyrnias et al., 2019), kidney disease (Liu et al., 2023), mitochondrial disease (Suarez-Rivero et al., 2022b), cancer (Inigo and Chandra, 2022) and neurodegenerative diseases (Beck et al., 2016; Cooper et al., 2017). For instance, in Alzheimer’s disease (AD), the two main toxic proteins that accumulate in AD, amyloid-β (Aβ) peptide and tau protein, impair mitochondrial function in the early stages of the disease (Torres et al., 2021; Bartman et al., 2024). Additionally, mitochondrial diseases are caused either by pathological mutations in mitochondrial DNA (mtDNA) or nuclear DNA affecting OXPHOS complexes, which are inherited maternally or in an autosomal recessive way, respectively (Gropman et al., 2024). In both cases, the UPR^mt^ is activated to compensate for mitochondrial dysfunction; however, at some points, this response is no longer enough to decrease mitochondrial damage (Suarez-Rivero et al., 2022a).

The activation of the UPR^mt^ has beneficial effects on increasing longevity (Xin et al., 2022) and improving mitochondrial function since it maintains ATP production, reduces ROS levels, and decreases apoptosis (Svagusa et al., 2020; Lu et al., 2022). Thus, it has been proposed that the activation of the UPR^mt^ could be a promising therapeutic approach for various diseases, although it seems paradoxical that inducing mitochondrial stress in the presence of mitochondrial dysfunction could be beneficial (Suarez-Rivero et al., 2022a). Antibiotics, mainly doxycycline, activate the UPR^mt^; however, the chronic use of antibiotics is still controversial (Suarez-Rivero et al., 2021). Therefore, recent studies have shown that different compounds could be safer therapeutic agents for several diseases (Table 1). Despite favorable outcomes, contradictory evidence indicates that overactivation or prolonged activation of the UPR^mt^ could be detrimental (Lu et al., 2022), indicating the importance of proper balance in the activation of the UPR^mt^.

**TABLE 1:** The UPR^mt^ in diseases.

Disease	UPR^mt^ state	Therapeutic approach	Beneficial effect	References
Mitochondrial disease	Active	Activation of the UPR^mt^ by - doxycycline - pterostilbene	❖ Restoration of normal mitochondrial protein expression patterns	Suarez-Rivero et al. (2022b), Suarez-Rivero et al. (2022)
			❖ Increase in complex I and IV activity	
			❖ Stabilization of mutated proteins to allow them to exert their function	
Neurodegenerative diseases	Active	Activation of the UPR^mt^ by - nicotinamide riboside - ginseng	❖ Reduction in Aβ levels and improvement of memory	Sorrentino et al. (2017), Liu et al. (2023), Zhou et al. (2020)
			❖ Increase in lifespan	
			❖ Increase in neurogenesis	
			❖ Rescue of neuronal loss	
Cardiac disease	Active	Activation of the UPR^mt^ by - nicotinamide riboside - tetrahydrocurcumin (THC)	❖ Reduction in cardiomyocyte death	Smyrnias et al. (2019), Zhang et al. (2020)
			❖ Attenuation of contractile dysfunction	
			❖ Attenuation of fibrosis	
Cancer	Active	Inhibition of individual UPR^mt^ components: dominant-negative ATF5 peptide - DCEM1 for Hsp60 - MKT077 for mtHsp70 - CDDO for Lonp1 - A2-32-01 for ClpP	❖ Decrease in the expression or activity of UPR^mt^-related proteins	Sun et al. (2020), Inigo et al. (2021), Kumar et al. (2022)
			❖ Reduction in cancer cell survival	
			❖ Reduction in cancer progression	

The activation of the UPR^mt^ is related to cancer progression (Keerthiga et al., 2021). ATF5, Hsp60, mtHsp70, Lonp1, and Clpp are upregulated in cancer, favoring tumor growth (Deng and Haynes, 2017; Inigo and Chandra, 2022). ATF5 induces the upregulation of antiapoptotic proteins such as Bcl-2 and MCL1, promoting tumor cell growth, and the upregulation of integrin-α2 and integrin-β1, which favors cancer cell invasion (Nukuda et al., 2016; Wang et al., 2022). Hsp60 is involved in preventing apoptosis by inhibiting mitochondrial permeability transition pore opening and stabilizing the protein survivin (Ghosh et al., 2010; Kim et al., 2019). mtHsp70 reduces p53 activity, promoting tumor cell survival, and regulates PI3K/AKT signaling to induce epithelial-mesenchymal transition of tumor cells (Wadhwa et al., 2002; Na et al., 2016). Lonp1 induces tumor metabolic reprogramming and promotes inflammatory cytokine production generating an immunosuppressive tumor environment (Quiros et al., 2014; Kuo et al., 2020). Finally, ClpP stabilizes OXPHOS complexes, maintaining ATP production, and regulates Src/PI3K/AKT signaling, favoring proliferation and invasion (Seo et al., 2016; Luo et al., 2020). Indeed, research on therapeutic approaches related to the UPR^mt^ in cancer have focused on the inhibition of the UPR^mt^, specifically on targeting individual UPR^mt^-associated proteins (Table 1) (Inigo et al., 2021).

## 5 UPR^mt^ regulation

Although the mechanism by which the UPR^mt^ is regulated is still not fully understood, some reports suggest that different proteins and signaling pathways could be involved in this process. In *C. elegans,* the SUMO protease ubiquitin-like protease 4 (ULP-4) regulates DVE-1 and ATFS-1 when the UPR^mt^ is induced. ULP-4 deSUMOylates DVE-1 to allow its accumulation in the nucleus, and deSUMOylates ATFS-1 to stabilize it and increases its transcriptional activity (Gao et al., 2019). These data suggest the posttranslational regulation of UPR^mt^-related transcription factors, which could also occur in mammals since, for example, ATF5 can also be SUMOylated and acetylated in other contexts (Liu et al., 2011; Yuan et al., 2018). However, this phenomenon has not yet been studied in mammals. In mammals, the protein GrpEL1, a nucleotide exchanger that controls the conversion of mtHsp70-ADP to mtHsp70-ATP, is also a regulator of the UPR^mt^. When this stress response is activated, GrpEL1 forms a complex with mtHsp70 to promote its function and reduce the aggregation of proteins in mitochondria (Ma et al., 2022). Additionally, a recent study suggested that the UPR^mt^ is linked to and dependent on mitophagy, with FUN14 domain-containing protein 1 (FUNDC1) acting upstream of its activation, inducing this stress response by decreasing the mtDNA content (Ji et al., 2022), which increases the misfolded protein load. Moreover, recently, it was shown that the activation of the UPR^mt^, in addition to the release of short DELE1 fragments, requires the release of mitochondrial ROS (mtROS) as signaling molecules into the cytosol (Sutandy et al., 2023). Once in the cytosol, mtROS oxidize the chaperone HSP40 (DNAJA1), which increases its interaction with cytosolic HSP70 to drive the translocation of HSF-1 to the nucleus to activate the transcription of mitochondrial chaperones and proteases (Sutandy et al., 2023).

Interestingly, in yeast, mitochondria trigger a UPR^mt^-like response before the UPR^mt^ is activated in response to mitochondrial precursor protein accumulation, which is an immediate response (Poveda-Huertes et al., 2020). This early response is mediated by the nuclear HMG-box domain-containing transcription factor Rox1, which translocates to mitochondria, maintaining mitochondrial import, the membrane potential, and translation (Poveda-Huertes et al., 2020). However, whether this early UPR^mt^-like response occurs in mammals or *C. elegans* is not known.

*Regarding signaling pathways,* there are some reports in *C. elegans* showing non-autonomous regulation through different pathways. One of these pathways is the follicle-stimulating hormone G protein-coupled receptor (FSHR1)/sphingosine kinase (SPHK-1) pathway, in which FSHR activates this stress response in neurons and promotes the stress-induced association of SPHK-1 with intestinal mitochondria (Kim and Sieburth, 2020). Additionally, ROS produced in GABAergic neurons act as signaling molecules by oxidizing the GABA_A_ receptor UNC-49 (Pohl et al., 2023). This oxidation of UNC-49 increases its channel activity in muscle cells, which induces the activation of the UPR^mt^ in intestinal cells via an unknown mechanism, suggesting that other tissues may be involved in the neuronal-intestinal regulation of the UPR^mt^ (Pohl et al., 2023). Moreover, it has been proposed that the Wnt signaling pathway may also be involved in the regulation of this stress response. Wnt signaling is a key pathway during development but is also important for proper adult neuronal function (Inestrosa et al., 2021). There are two pathways of Wnt signaling, the β-catenin-independent or non-canonical signaling and β-catenin-dependent or canonical signaling pathway, which regulate the expression of Wnt target genes (Inestrosa and Arenas, 2010; Inestrosa et al., 2021). Preliminary results from our laboratory indicate that mitochondrial chaperones and proteases involved in the UPR^mt^ have Wnt-responsive elements in their promoters (Torres et al., 2022a; b), and the modulation of Wnt signaling, both in *C. elegans* and in primary hippocampal neuronal culture, regulates the expression of UPR^mt^-associated proteins (Torres et al., 2022a; b; Torres et al., 2023). These data suggest that Wnt signaling may have a direct effect on the expression of UPR^mt^ genes, which could be Wnt target genes.

## 6 Conclusion

The UPR^mt^, which is involved in the mitochondrial stress response, is a key signaling pathway for maintaining the protective function of mitochondria upon protein accumulation. This stress response has been described in yeast, nematodes, and mammals, suggesting that it is an essential protective mechanism for survival among eukaryotic organisms. Indeed, it has been described as a compensatory response that reduces mitochondrial damage in several diseases; however, at some point, the degree of mitochondrial dysfunction reaches a critical level, and endogenous activation of the UPR^mt^ is insufficient for countering it. Although the mechanism underlying the UPR^mt^ has been described over the years, the regulation of this stress response has been less studied. Thus, more information about how to safely modulate the UPR^mt^ while avoiding the detrimental effects that could result from its long-term activation is needed. This information is essential for the development of new drug-based therapeutic approaches for chronic diseases such as mitochondrial diseases, cancer, and AD.
